# Evaluation of the Efficacy of UV-C Radiation in Eliminating *Clostridioides difficile* from Touch Surfaces Under Laboratory Conditions

**DOI:** 10.3390/microorganisms13050986

**Published:** 2025-04-25

**Authors:** Anna Różańska, Anna Pioskowik, Laura Herrles, Tanisha Datta, Paweł Krzyściak, Estera Jachowicz-Matczak, Tomasz Siewierski, Monika Walkowicz, Agnieszka Chmielarczyk

**Affiliations:** 1 Department of Microbiology, Faculty of Medicine, Jagiellonian University Medical College, 31-008 Kraków, Poland; pawel.krzysciak@uj.edu.pl (P.K.); estera.jachowicz-matczak@uj.edu.pl (E.J.-M.); agnieszka.chmielarczyk@uj.edu.pl (A.C.); 2 Students’ Scientific Group of Microbiology, Faculty of Medicine, Jagiellonian University Medical College, 31-008 Kraków, Poland; ania.pioskowik@student.uj.edu.pl (A.P.); laura.herrles@student.uj.edu.pl (L.H.); tanisha.datta@student.uj.edu.pl (T.D.); 3 St. Rose Hospital, ul. Skotnicka 230 A, 30-394 Kraków, Poland; tsiewierski@outlook.com; 4 AGH University of Krakow, Faculty of Non-Ferrous Metals, al. Mickiewicza 30, 30-059 Kraków, Poland; mwa@agh.edu.pl

**Keywords:** *C. difficile*, UV-C disinfection, hospital infections

## Abstract

*Clostridioides difficile* (CD) is a Gram-positive, spore-forming anaerobic bacterium, usually transmitted through the fecal–oral route, that can result from direct person-to-person contact, exposure to contaminated environmental surfaces, or contact with the hands of colonized healthcare personnel. An increased number of infections, especially healthcare-associated, with this etiology has been observed in most countries. As a spore-forming organism, CD is resistant to alcohol formulations and is a challenge for chemical disinfection. The solution could be the supplementation of traditional disinfection with non-touch techniques, such as UV-C radiation. The adoption of UV-C as a supplementary disinfection method in hospitals has significantly increased since the COVID-19 pandemic. However, there are no current guidelines concerning the use of UV-C disinfection in hospitals. The aim of this study was to evaluate the effectiveness of UV-C irradiation in inactivating *Clostridioides difficile* from different types of surfaces in hospital settings. The study was based on laboratory tests evaluating the efficacy in eliminating three different *C. difficile* strains on carriers made of plastic, metal and glass after 10 min exposure to UV-C (wavelength, 253.7 nm). We observed a wide range of reductions in the *C. difficile* suspensions depending on the density of the carrier contamination, type of carrier, strains and the location of the carrier. The percentage reductions ranged from 0 to 100%, but the best results were observed for glass, with lower initial suspension density and carrier placement on a door frame. Statistically significant differences were only seen in different suspension densities. Our experiment was a continuation of the tests done for non-sporing bacteria and *C. auris*, and there were some interesting differences in *C. difficile* reflecting its biology, especially its sensitivity to an aerobic atmosphere during the sample drying. Although the elimination of *C. difficile* by UV-C radiation was confirmed in our experiment, it was lower than in the case of non-spore-forming bacteria. Thus, this method may be used in healthcare settings (hospitals) for improving environmental safety and preventing *C. difficile* spreading.

## 1. Introduction

*Clostridioides difficile* is a Gram-positive, spore-forming anaerobic bacterium, usually transmitted through the fecal–oral route, that can result from direct person-to-person contact, exposure to contaminated environmental surfaces or contact with the hands of colonized healthcare personnel [1]. According to the Centers for Disease Control (CDC) in the United States, the bacterium infects half a million Americans every year. Based on the severity of the infections, approximately 29,000 patients had severe outcomes within the first month of the infection, and of these patients, 15,000 deaths were directly associated with *C. difficile*. The epidemiology regarding *C. difficile* infections (CDI) has evolved within the past 2 decades [2]. In Poland specifically, CD infections more than doubled during the years 2013–2018 [3]. In the years 2016–2017, healthcare-associated CDI incidence in Poland reported by the European Healthcare-associated infections-Net (HAI-Net) project was 6.18/10,000 patient days (ptds), and it was more than twice as high as the average 2.87/10,000 ptds for the countries included in the project. The incidence rates were higher only in Estonia and Lithuania [4]. In a later, albeit single-center study, the incidence of *C. difficile* was 55.4/100,000 people, which is close to the ECDC project findings [5] The reasons for such a high incidence may be a lack of effective implementation of antimicrobial stewardship programs and several problems with effective implementation of infection control and prevention. The high HAI incidence rate in important patient populations, such as intensive care units and surgical procedures [6,7], is one of the facts confirming this. Additionally, antibiotic consumption in Polish hospitals reported by some of the latest studies is higher than expected [8,9], and compliance with hand hygiene recommendations is unsatisfactory [10,11]. *C. difficile* infections in hospitals are mainly a consequence of the overuse of antibiotics and a lack of effective implementation of infection control and prevention. Since the bacterium can form spores, and these spores can survive outside the human body, often for months at a time, they are very resistant to disinfectants and chemical substances, leading to higher rates of infection [1]. Also, there is a need to search for supplementary solutions to prevent CDI. One could be the implementation of additional disinfection techniques, such as UV-C. Such additional non-touch disinfection may be necessary due to personnel-related variability in cleaning adequacy and protocol and disinfectant-based variability, which can severely impact the effectiveness of traditional chemical disinfection.

UV light can be used as a disinfectant in a process called ultraviolet germicidal irradiation (UVGI). UV irradiation has a wavelength of 100–400 nm and can be further classified into vacuum UV (100–200 nm), UV-A (315–400 nm), UV-B (280–320 nm), and UV-C (200–280 nm) [12]. The range of 250–270 nm is known as the germicidal spectrum, with 256 nm characterized by the strongest absorption by the DNA of microorganisms and their inactivation [13].

When bacterial spores are treated with UV-C radiation, DNA absorbs photons and induces the formation of bipyrimidine dimers, especially spore photoproducts. These transiently block DNA transcription and replication, leading to cell death [14,15].

The adoption of UV-C as a supplementary disinfection method in hospitals has significantly increased since the COVID-19 pandemic. However, there are no current guidelines concerning the use of UV-C disinfection in hospitals. Additionally, Resendis et al. [16], in a review study covering over 180 articles, stated in a 2023 analysis that while several reviews have been dedicated to evaluating the effectiveness of enhanced “no-touch” disinfection, including ultraviolet intervention, none to their knowledge have focused on characterizing surface-specific effectiveness of UV-C. The authors of this study claimed that due to the known influence of variables such as the frequency of touch, porosity of the material, and obstruction of the UV light path, it is important to profile potential vectors in the patient environment more precisely, aiming to identify surfaces/areas that may require enhanced treatment [16].

The aim of this study was to evaluate the effectiveness of UV-C irradiation in the elimination of *Clostridioides difficile* isolated from Polish hospitals, suspended on different types of surfaces commonly used in hospital settings.

## 2. Materials and Methods

**Figure 1:**
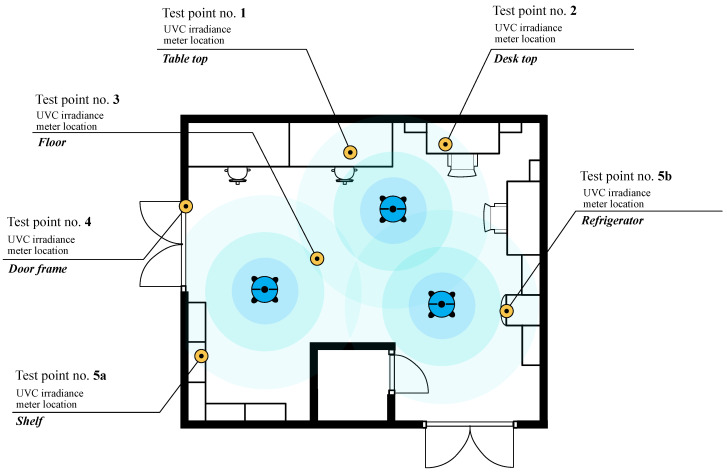
Plan of the room where the effectiveness of surface disinfection by the UV-C method using OCTA robots was tested.

The study, performed between 22 May 2023 and 30 June 2023, employed a method based on the contamination of carriers made of various materials with a specific volume. A bacterial suspension of known density was subjected to UV-C disinfection. Before coating the materials, the bacteria were subjected to a temperature shock, which promotes spore production in *C. difficile*. Three types of test carriers cut into squares with 2 cm sides were used: stainless steel (metal), plastic and glass. The materials that the test plates (carriers) were made of were selected in terms of representativeness as regards hospital equipment of various types. Three different *C. difficile* strains were used for the study (002, 014 and hypervirulent 027 ribotype strains). Strain 027 was selected due to its hypervirulent nature, producing higher amounts of toxins compared to other strains. Additionally, this strain exhibits increased resistance to antibiotics, which is a crucial aspect in CDI treatment. Strain 027 is also the most frequently occurring strain in Poland [16]. In comparison, strain 014 was chosen as it is commonly isolated from the pediatric population, suggesting it is also a circulating strain [17]. As a third strain in the study, strain 002 was selected, which is not frequently mentioned in the literature as a common isolate in Poland. This makes it an interesting control case for comparison with other, more commonly occurring strains. All the strains came from the collection of the Department of Microbiology of the Jagiellonian University Medical College and were isolated from infections in hospitalized patients. We used the OCTA UV-System (Eco-Light Biosafety Ltd., Słupsk, Poland) as the source of UV-C, with a wavelength of 253.7 nm and a device operating time of 10 min. The OCTA UV-System is a set of three lamps emitting UV-C radiation. The tests were carried out in a room with an area of 27.45 m^2^ (room height 3.8 m). The distance between the lamps and the carriers located on the floor, desk, door frame, refrigerator and table top was around 1.5 m. The location of the carriers in the room is shown in Figure 1.

Before the experiment, for the given testing points, the doses of UV-C were measured by the supplier of the OCTA UV-C with the SONOPAN UVC-2GRM radiometer; the results are presented in Table 1. During testing, the colorimetric Tri Card UVC dosimeters (Intellego Technologies, Stockholm, Sweden) were used to confirm the expected dosages for all the testing points.

The detailed procedure for assessing the effectiveness of *Clostridioides difficile* elimination was as follows:

1. The strain *C. difficile* was inoculated into 10 mL of BHI (brain–heart infusion) broth and then cultured under anaerobic conditions at 37 °C for 24 h. The following day, the *C. difficile* cultures remained under microaerophilic conditions at room temperature for 24 hours to allow spore germination, and then were diluted in BHI broth to a density of 0.5 McF (density A), which is equal to 10^8^ CFU/mL.
2. Culture A was diluted in order to obtain density B, equal to 10^7^ CFU/mL.
3. The cultures of known density (density A or density B, depending on the experiment option) were transferred onto metal, glass and plastic carriers in a volume of 50 µL. These were left to dry (which took about 1.5 h), and then the samples distributed over five test points (table top floor, desk top, refrigerator/shelf, door frame) were subjected to UV disinfection (OCTA-UV C Multisystem). Following 10 min of disinfection, samples from the tested materials were collected using flocked swabs.
4. Subsequently, a serial dilution in saline was carried out, and 100 μL specimens were seeded onto blood agar plates using the spread plate method.
5. The control consisted of metal, glass and plastic substrates with the appropriate strain applied, untreated with UV light.
6. The initial bacterial suspension was also inoculated to determine its exact density (CFU/mL). The samples were incubated for 4 days under anaerobic conditions at 37 °C.

The results are presented in terms of the absolute and percentage reductions of bacterial cells.

### Statistical Analysis

Monovariate comparisons of microorganism reduction percentages were performed using the Wilcoxon rank sum test for two bacterial suspensions and the Kruskal–Wallis test for material and strain groups. A Kruskal–Wallis test was also applied to assess the interaction between the material and bacterial suspensions. All the tests were conducted in RStudio 2024.12.1 with R version 4.4.3 (28 February 2025) with a significance level of 0.05.

## 3. Results

The results show the degree of reduction in the tested *C. difficile* strains depending on the type of carrier, i.e., metal, plastic or glass; their locations and strains are presented in Table 2 and Table 3. The results for the density A (10^8^ CFU/mL) samples are presented in Table 1, while those for density B (10^7^ CFU/mL) are in Table 2.

For both options of the experiment (densities A and B), the data on the control density are also given in Table 2 and Table 3. The control samples were the carriers (metal, plastic and glass) contaminated by test strains and not exposed to UV-C. The control sample densities (assessed after recovery from the carriers at the same time from contamination as the test samples) were four to five logarithms lower than the suspension densities (A or B) obtained directly from the solutions prepared for carrier contamination (test and control carriers). For density A, the control sample densities ranged from 3.40 × 10^3^ to 3.00 × 10^2^, and for density B, from 1.20 × 10^4^ to 2.00 × 10^2^, depending on the type of carrier and strain (Table 2 and Table 3).

**Figure 2:**
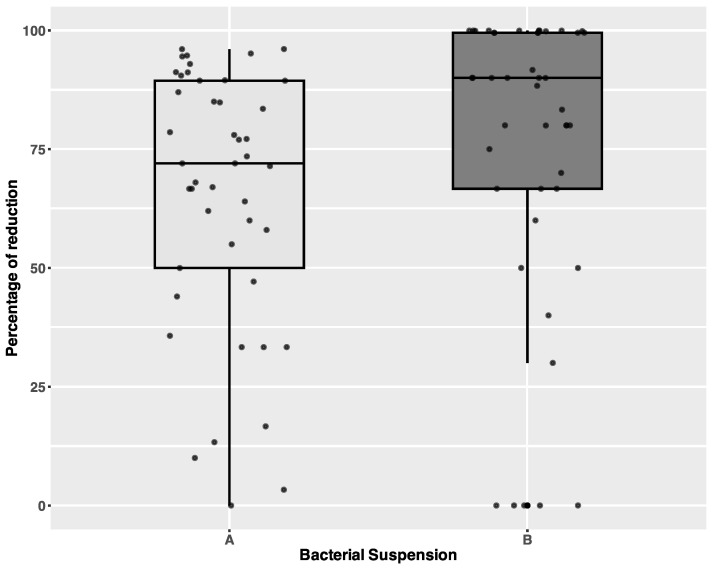
Differences in percentage of reduction across different density categories. Individual data points are shown with jitter. A—density of bacterial suspension 10^8^ CFU/mL, B—density of bacterial suspension 10^7^ CFU/mL.

Statistically significant differences in the levels of reduction were observed between densities A and B. A higher level of percentage reduction was observed in the case of density B (median A = 72%, B = 90%, average A = 65%,3, B = 75%) (Wilcoxon rank sum test with continuity: W = 689, *p*-value = 0.009086). The results are presented in Figure 2.

For types of carriers, the highest, although not statistically significant, level of reduction was observed for glass (Kruskal–Wallis chi-squared = 3.3125, df = 2, *p*-value = 0.1909), Figure 3.

**Figure 4:**
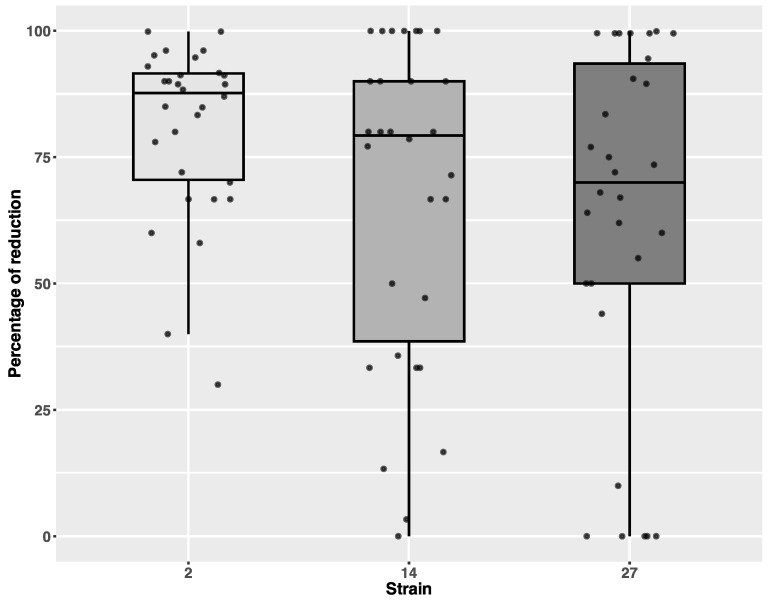
Differences in percentage of reduction depending on the *C. difficile* strain. Individual data points are shown with jitter.

Different, although not significant, levels of reduction were also observed between all the tested strains of *C. difficile* (Kruskal–Wallis chi-squared = 3.0476, df = 2, *p*-value = 0.2179), Figure 4.

For the location of the carriers, the highest level of reduction (not significant) was observed for the door frame (Kruskal–Wallis chi-squared = 5.8983, df = 4, *p*-value = 0.2069), Figure 5.

## 4. Discussion

The presented study on the effectiveness of UV-C radiation in eliminating *C. difficile* was a continuation of the study against bacteria from the ESKAPE group and *C. auris* [18]. For this study for *C. difficile*, the same method was used, but it turned out that after two hours of drying of the suspension on carriers in the laboratory room, the density of control samples for *C. difficile* ranged from approx. 10^2^–10^3^, while for the remaining non-sporing bacteria and for *C. auris*, the density on control media was around 10^6^.

In the case of non-sporing bacteria and *C. auris*, the difference compared to the suspension applied to carriers of approximately 10^7^ was due to the loss associated with the recovery method (swab). In the case of *C. difficile*, the difference of 4–5 logarithms compared to the initial suspension was related to two hours of drying in aerobic conditions and reflects a decrease in the number of vegetative *C. difficile*. It can therefore be assumed that this exposure did not result in a decrease in the number of spores, and therefore, the method can be considered a good reflection of the actual conditions of *C. difficile* contamination in the hospital environment. In our study, materials inoculated with *C. difficile* were stored under aerobic conditions both prior to and during UV-C irradiation. This approach aimed to preserve the viability of the spores, as *C. difficile* is an obligate anaerobe and its spores are notably resilient to environmental stresses. Our findings indicate that desiccation (drying) effectively eliminated the vegetative forms of *C. difficile*. Subsequent exposure to UV-C light primarily targeted the bacterial spores. This observation aligns with previous research demonstrating that UV-C irradiation can significantly reduce *C. difficile* spore viability [19].

The observed reduction on all types of carriers, in all locations and for all tested *C. difficile* strains was lower than in the case of non-sporing bacteria and *C. auris* [18]. The results indicate that the reduction in *C. difficile* density depends on many factors. Evaluating the results collectively, the best reduction effect was achieved on glass samples, especially at the initial suspension density B. This observation is consistent with previous studies on other bacterial species [18]. However, significant differences in reduction were observed when comparing individual results obtained for specific strains, as well as results for individual sample locations. The differences between the strains from the same species were observed even for non-sporing and easy-to-culture bacteria and different non-touch disinfection methods [19]. *C. difficile* is notoriously difficult to isolate, cultivate and extremely sensitive to oxygen, and the process effectiveness could be interfered with by many factors, for example, shadowing or spore clumping. This might be the fact in our study, where for suspension B of the hypervirulent, and with biofilm-forming ability strain 027, we observed a surprisingly wide range of reductions depending on the location of the carrier, especially metal ones. Thus, the research should be supplemented with tests with a larger number of repetitions and/or a larger number of strains, despite the fact that there have been several studies investigating the effect of UV-C disinfection focused on *C. difficile*. Koutras et al. showed an overall 98.17% reduction in spores of *C. difficile* after UV-C disinfection [20]. The laboratory-based study of Blau and Gallert revealed substantial differences between five tested CD strains in a 10-minute exposure time [21]. They also proved significant differences for exposure time (20 min and more exposure were highly effective, but may be less convenient in practice), the suspensions of the tested strains and the doses of UV-C per cm^−2^ (the highest efficacy of reduction for doses more than 2700 mj per cm^−2^ ) [21]. Liscynesky et al. also observed that routine use of UV light disinfection significantly reduces the number of *C. difficile* isolated from hospital surfaces, suggesting that appropriate exposure time may also affect spores of the microorganism [22].

As mentioned above, our experiment showed high, on average, percentages in the reduction of *C. difficile* after 10 min exposure to UV-C. The level of reduction expressed in the decrease in number of logarithms, however, does not meet the European normative requirements. For the medical sector, in both suspension and surface disinfection, indicate a ≥5 log_10_ reduction in CFU as a threshold for successful disinfection [23]. However, our bacterial suspensions used for the experiment were much higher than surfaces not visibly contaminated after traditional decontamination in hospitals. In addition, we confirmed more effective sporicidal properties of UV-C in the case of lower initial density (density A vs. B). Thus, our results confirm the conclusion of Casini et al. that UV-C radiation can be significantly more successful when performed together with manual disinfection than manual disinfection of hospital surfaces done alone [24]. Additionally, the cost and user-friendliness of UV-C light are still being assessed but suggest it is efficient and easy for hospitals to use. UV-C also allows hospitals to disinfect a whole room at once, making it highly efficient [25]. Ploydaeng et al. confirm the advantage of UV-C as an adjunctive procedure to the standard cleaning method with respect to its safety [26]. They found that no studies conclusively confirm the positive correlation between short-term radiation of 254 nm UV-C light and skin cancer, although chronic radiation may cause skin erythema, desquamation, and abnormal histological findings. Therefore, avoiding chronic and direct exposure to UVC light is a safety requirement.

The next step after laboratory testing of the effectiveness of UV-C disinfection is research on its influence on the rates and financial impact of healthcare-associated infections. One such study is that of Raggi et al., which shows a decrease in HAIs rates in a period with additional UV-C disinfection accompanied by a significant reduction in costs [27]. However, the cited study did not take into account CD infections, but only several of the most common infections due to non-sporing bacteria. However, Maugeri et al.’s review, published in 2025, found four studies focused on the impact of UV-C irradiation on rates of CDI [28]. In three of them, the authors showed the reduction in *C. difficile* infection rates, and in one of these, no such effect was observed [28].

The core element for implementing this method in routine practice could be setting the optimal exposure (disinfection) time, also due to practical reasons. We tested a 10 min exposure, which could probably be quick enough and effective, as our results show. The direct UV-C irradiation may be used only in rooms with no patients present, so simplicity and quick action are important. This method is mainly being considered for acute care hospitals, but gastrointestinal tract infections, including those with *C. difficile* etiology, are also common in long-term care facilities (LTCFs). LTCFs are struggling with shortages of medical and care staff, so, in case of outbreaks, the usage of quick and simple disinfection may be valuable. We mentioned that the level of logarithmic reduction was not as high as recommended by European standards, but we should remember that environmental contamination is often much lower than the starting suspension density in our experiment, and UV-C disinfection is only an option for supplementing of traditional chemical disinfection. For proven contamination of highly virulent strains, a longer exposure time (20 min at least) should be considered.

Limitations in the study: This study has some limitations, such as a limited number of tested strains and types of carriers. Fabrics are also common in healthcare settings; however, mainly linens, gowns and surgical drapes can be washed and disinfected with thermal methods or even sterilized; these were not the target of our study due to limited resources. Additionally, the doses of the UV-C measured once before the experiment should be treated as indicative. However, our study proves the efficacy of UV-C irradiation in laboratory conditions simulating real environments and can have an important impact in the frame of future research. According to Maugeri et al., future research should prioritize large-scale, randomized controlled trials to provide stronger and reliable evidence of the efficacy of UV-C technologies [28]. Additionally, research should focus on evaluating the cost-effectiveness of these systems, as financial feasibility will be a critical factor in their widespread adoption, because professional machines in this technology are of substantial cost. Standardized, long-term investigations are essential to better understand the practical impact of UV-C disinfection on reducing HAIs and to establish evidence-based guidelines for its implementation in clinical practice.

## Figures and Tables

**Figure 3:**
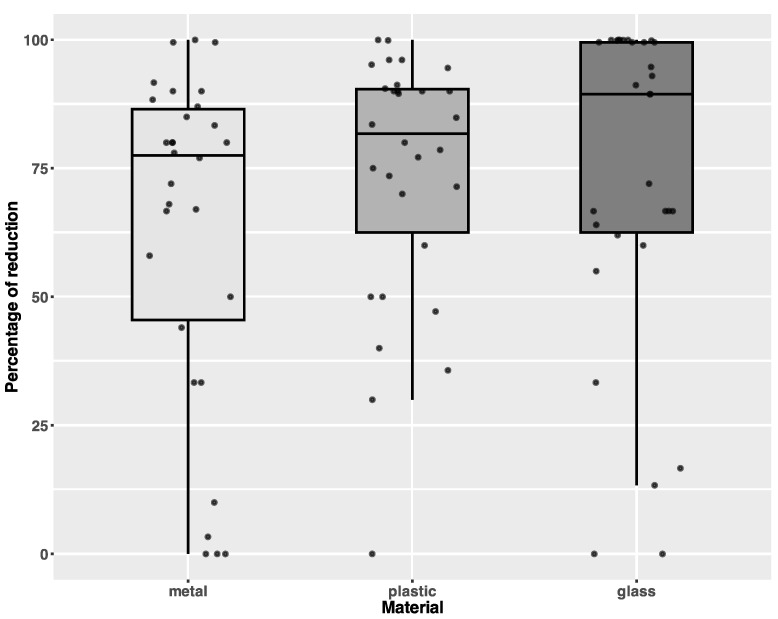
Differences in percentage of reduction depending on the type of carrier. Individual data points are shown with jitter.

**Figure 5:**
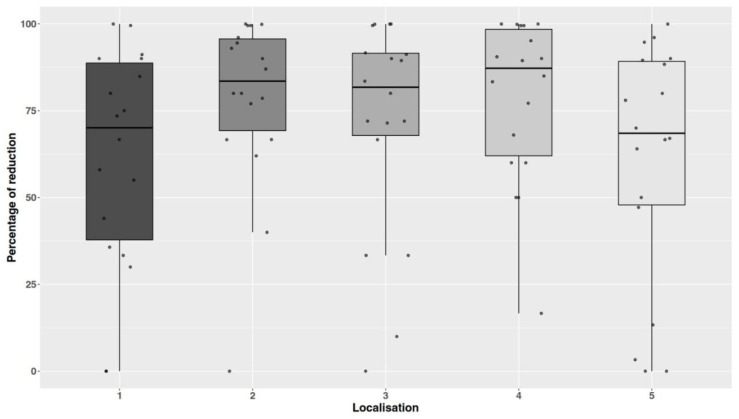
Differences in percentage of reduction depending on the locations of the carriers. Individual data points are shown with jitter. Legend: 1—table top, 2—a desk top, 3—floor, 4—door frame, 5—Refrigerator/shelf.

**Table 1:** Average values of doses of UV-C [mJ/cm^2^] for given testing locations in the room used for the study on the efficacy of disinfection with the OCTA UV-C Multisystem.

Testing Points	10 min
Testing point no. 1	472 mJ/cm^2^
Testing point no. 2	273 mJ/cm^2^
Testing point no. 3	542 mJ/cm^2^
Testing point no. 4	925 mJ/cm^2^
Testing point no. 5a	636 mJ/cm^2^
Testing point no. 5b	1059 mJ/cm^2^

**Table 2:** Reduction in tested strains of density A according to the type of carrier and location.

	Metal			Plastic			Glass		
**Strain 002**									
**Location**	(cfu/mL)	reduction log	% of reduction	(cfu/mL)	reduction log	% reduction	(cfu/mL)	reduction log	% reduction
**1**	4.20 × 10^2^	5.80 × 10^2^	58.00	5.00 × 10^2^	2.80 × 10^3^	84.85	3.00 × 10^2^	3.10 × 10^3^	91.18
**2**	1.30 × 10^2^	8.70 × 10^2^	87.00	1.30 × 10^2^	3.17 × 10^3^	96.06	2.40 × 10^2^	3.16 × 10^3^	92.94
**3**	2.80 × 10^2^	7.20 × 10^2^	72.00	2.90 × 10^2^	3.01 × 10^3^	91.21	3.60 × 10^2^	3.04 × 10^3^	89.41
**4**	1.50 × 10^2^	8.50 × 10^2^	85.00	1.60 × 10^2^	3.14 × 10^3^	95.15	3.60 × 10^2^	3.04 × 10^3^	89.41
**5**	2.20 × 10^2^	7.80 × 10^2^	78.00	1.30 × 10^2^	3.17 × 10^3^	96.06	1.80 × 10^2^	3.22 × 10^3^	94.71
**Control**	1.00 × 10^3^			3.30 × 10^3^			3.40 × 10^3^		
**Strain 014**									
**1**	4.00 × 10^2^	2.00 × 10^2^	33.33	4.50 × 10^2^	2.50 × 10^2^	35.71	3.00 × 10^2^	0.00 × 10^0^	0.00
**2**	2.00 × 10^2^	4.00 × 10^2^	66.67	1.50 × 10^2^	5.50 × 10^2^	78.57	1.00 × 10^2^	2.00 × 10^2^	66.67
**3**	4.00 × 10^2^	2.00 × 10^2^	33.33	2.00 × 10^2^	5.00 × 10^2^	71.43	2.00 × 10^2^	1.00 × 10^2^	33.33
**4**	3.00 × 10^2^	3.00 × 10^2^	50.00	1.60 × 10^2^	5.40 × 10^2^	77.14	2.50 × 10^2^	5.00 × 10^1^	16.67
**5**	5.80 × 10^2^	2.00 × 10^1^	3.33	3.70 × 10^2^	3.30 × 10^2^	47.14	2.60 × 10^2^	4.00 × 10^1^	13.33
**Control**	6.00 × 10^2^			7.00 × 10^2^			3.00 × 10^2^		
**Strain 027**									
**1**	5.60 × 10^2^	4.40 × 10^2^	44.00	5.30 × 10^2^	1.47 × 10^3^	73.50	4.50 × 10^2^	5.50 × 10^2^	55.00
**2**	2.30 × 10^2^	7.70 × 10^2^	77.00	1.10 × 10^2^	1.89 × 10^3^	94.50	3.80 × 10^2^	6.20 × 10^2^	62.00
**3**	9.00 × 10^2^	1.00 × 10^2^	10.00	3.30 × 10^2^	1.67 × 10^3^	83.50	2.80 × 10^2^	7.20 × 10^2^	72.00
**4**	3.20 × 10^2^	6.80 × 10^2^	68.00	1.90 × 10^2^	1.81 × 10^3^	90.50	4.00 × 10^2^	6.00 × 10^2^	60.00
**5**	3.30 × 10^2^	6.70 × 10^2^	67.00	2.10 × 10^2^	1.79 × 10^3^	89.50	3.60 × 10^2^	6.40 × 10^2^	64.00
**Control**	1.00 × 10^3^			2.00 × 10^3^			1.00 × 10^3^		

Legend: 1—table top, 2—a desk top, 3—floor, 4—door frame, 5—Refrigerator/shelf.

**Table 3:** Reduction in tested strains of density B according to the type of carrier and location.

	Metal			Plastic			Glass		
**Strain 002**									
**Location**	(cfu/mL)	reduction log	% of reduction	(cfu/mL)	reduction log	% reduction	(cfu/mL)	reduction log	% reduction
**1**	1.20 × 10^3^	1.00 × 10^4^	90.00	1.40 × 10^3^	6.00 × 10^2^	30.00	2.00 × 10^2^	1.00 × 10^2^	66.70
**2**	2.40 × 10^3^	9.60 × 10^3^	80.00	1.20 × 10^3^	8.00 × 10^2^	40.00	0.00 × 10^0^	6.00 × 10^2^	100.00
**3**	1.00 × 10^3^	1.10 × 10^4^	91.70	2.00 × 10^2^	1.80 × 10^3^	90.00	2.00 × 10^2^	4.00 × 10^2^	66.70
**4**	2.00 × 10^3^	1.00 × 10^4^	83.30	8.00 × 10^2^	1.20 × 10^3^	60.00	0.00 × 10^0^	6.00 × 10^2^	100.00
**5**	1.40 × 10^3^	1.06 × 10^4^	83.30	6.00 × 10^2^	1.40 × 10^3^	70.00	2.00 × 10^2^	4.00 × 10^2^	66.70
**Control**	1.20 × 10^4^			2.00 × 10^3^			6.00 × 10^2^		
**Strain 014**									
**1**	4.00 × 10^2^	1.60 × 10^3^	80.00	2.00 × 10^2^	1.80 × 10^3^	90.0	0.00 × 10^0^	2.00 × 10^3^	100.00
**2**	4.00 × 10^2^	1.60 × 10^3^	80.00	2.00 × 10^2^	1.80 × 10^3^	90.0	0.00 × 10^0^	2.00 × 10^3^	100.00
**3**	0.00 × 10^0^	2.00 × 10^3^	100.00	4.00 × 10^2^	1.60 × 10^3^	80.0	0.00 × 10^0^	2.00 × 10^3^	100.00
**4**	2.00 × 10^2^	1.80 × 10^3^	90.00	0.00 × 10^0^	1.60 × 10^3^	100.0	0.00 × 10^0^	2.00 × 10^3^	100.00
**5**	4.00 × 10^2^	1.60 × 10^3^	80.00	2.00 × 10^2^	1.80 × 10^3^	90.0	0.00 × 10^0^	2.00 × 10^3^	100.00
**Control**	2.00 × 10^3^			2.00 × 10^3^			2.00 × 10^3^		
**Strain 027**									
**1**	2.00 × 10^2^	0.00 × 10^0^	0.00	2.00 × 10^2^	6.00 × 10^2^	75.00	0.00 × 10^0^	2.00 × 10^2^	100.00
**2**	0.00 × 10^0^	2.00 × 10^2^	100.00	8.00 × 10^2^	0.00 × 10^0^	0.00	0.00 × 10^0^	2.00 × 10^2^	100.00
**3**	2.00 × 10^2^	0.00 × 10^0^	0.00	0.00 × 10^0^	8.00 × 10^2^	100.00	0.00 × 10^0^	2.00 × 10^2^	100.00
**4**	0.00 × 10^0^	2.00 × 10^2^	100.00	4.00 × 10^2^	4.00 × 10^2^	50.00	0.00 × 10^0^	2.00 × 10^2^	100.00
**5**	2.00 × 10^2^	0.00 × 10^0^	0.00	4.00 × 10^2^	4.00 × 10^2^	50.00	2.00 × 10^2^	0.00 × 10^0^	0.00
**Control**	2.00 × 10^2^			8.00 × 10^2^			8.00 × 10^2^		

Legend: 1—table top, 2—a desk top, 3—floor, 4—door frame, 5—Refrigerator/shelf.

## Data Availability

The data presented in this study are available on request from the corresponding author.
